# Supplementary material for: Evaluation of the Efficacy of Immune and Inflammatory Markers in the Diagnosis of Lacrimal-Gland Benign Lymphoepithelial Lesion
Source: Curr Issues Mol Biol. 2023 Mar 1;45(3):2013–20. doi: 10.3390/cimb45030129 (PMC10047269; doi:10.3390/cimb45030129)
Supplement: Supplementary file 1 [file cimb-45-00129-s001.zip › supplement material.pdf]

Supplementary Figure S1.

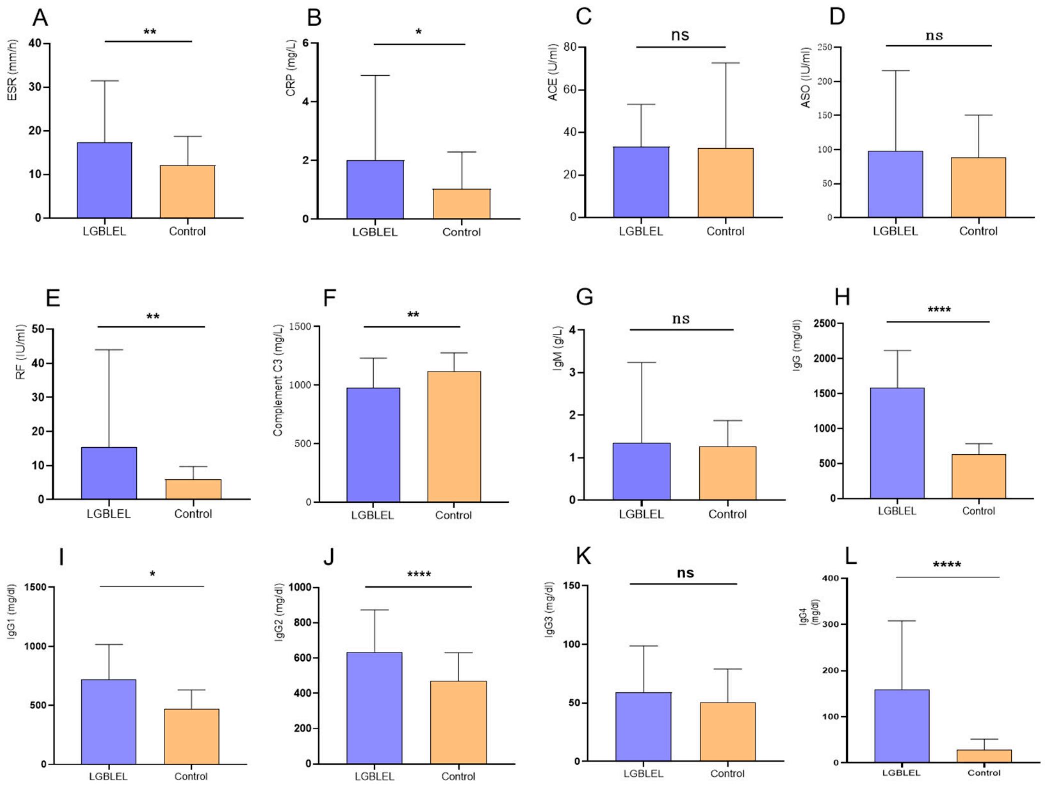

Supplementary Figure S1. Comparative analysis images of immune and inflammatory indicators between LGBLEL and control groups. Comparatively analyse the levels of ESR (A), CRP (B), ACE(C), ASO(D), RF(E), Complement C3(F), IgM(G), IgG(H), IgG1(I), IgG2(J), IgG3(K), IgG4(L) in peripheral blood between LGBLELgroup and control group, separately. "ns" refers to no significant difference (P>0.05), "*" refers to P ＜ 0.05, "**" refers to P ＜ 0.01, "****" refers to P ＜ 0.0001.

Supplementary Table S1. Multifactor Logistic Regression Analysis of Other Differential Indicators between the LGBLEL patients and the Control Group

Indicators	Wald-value	*P* value	Correlation	95% confidence interval	
				Lower limit	Upper limit
ESR	0.029	0.865	0.977	0.748	1.276
CRP	3.023	0.082	4.403	0.828	23.414
RF	0.064	0.800	1.065	0.655	1.732
IgG1	0.003	0.959	1.000	0.991	1.008
IgG2	2.631	0.105	1.007	0.999	1.016
Constant	1.369	0.242	0.061	-	-

Abbreviation：ESR, erythrocyte sedimentation rate; CRP, C-reactive protein; RF, rheumatoid factor ; IgG1, immunoglobulin G1; IgG2, immunoglobulin G2.
